# A Spindle Cell Tumor in Disguise: Histoid Nodules Arising From Peripheral Nerves

**DOI:** 10.7759/cureus.86173

**Published:** 2025-06-16

**Authors:** Akshdeep Singh Narula, Jerene Mathews, Pratik Minj, Ankur Patel

**Affiliations:** 1 Department of Dermatology, All India Institute of Medical Sciences, Bhopal, Bhopal, IND; 2 Department of Pathology and Laboratory Medicine, All India Institute of Medical Sciences, Bhopal, Bhopal, IND; 3 Department of Radiodiagnosis, All India Institute of Medical Sciences, Bhopal, Bhopal, IND

**Keywords:** acid fast bacilli, histiocytes, histoid leprosy, mycobacterium leprae, nerve, peripheral nerve lesion, spindle cell, storiform pattern, subcutaneous nodules, ultrasound

## Abstract

A 25-year-old male presented with multiple, painless subcutaneous nodules, each measuring 5-6 mm, attached to thickened superficial cutaneous nerves. He also had nodular infiltration of the left earlobe. Histopathology revealed spindle-shaped histiocytes in a storiform pattern, with numerous acid-fast bacilli in Fite-Faraco staining. Skin slit smear from the nodule revealed a bacteriological index of +6. Ultrasonography of the nodule revealed a well-defined, avascular, heterogenous hypoechoic lesion over a focally thickened nerve with loss of fibrillary pattern, confirming a rare form of neural histoid leprosy.

## Introduction

Histoid leprosy is an unusual presentation of leprosy with high bacillary load, which can be a reservoir for infection [1]. It can be challenging to recognize, as atypical forms can present without the cardinal signs of leprosy, which include three features: hypopigmented or erythematous skin lesions with the loss of sensation, the involvement of peripheral nerves in the form of definite thickening with sensory impairment, and skin smear positive acid-fast bacilli (AFB) [2]. A growing body of literature discusses atypical manifestations of histoid leprosy. Our case introduces a rare and biologically unique feature: histoid nodules arising directly from peripheral nerves. It seems plausible from our case that histoid nodules might arise from the redifferentiation of Schwann cells into mesenchymal cells.

## Case presentation

**Figure 1:**
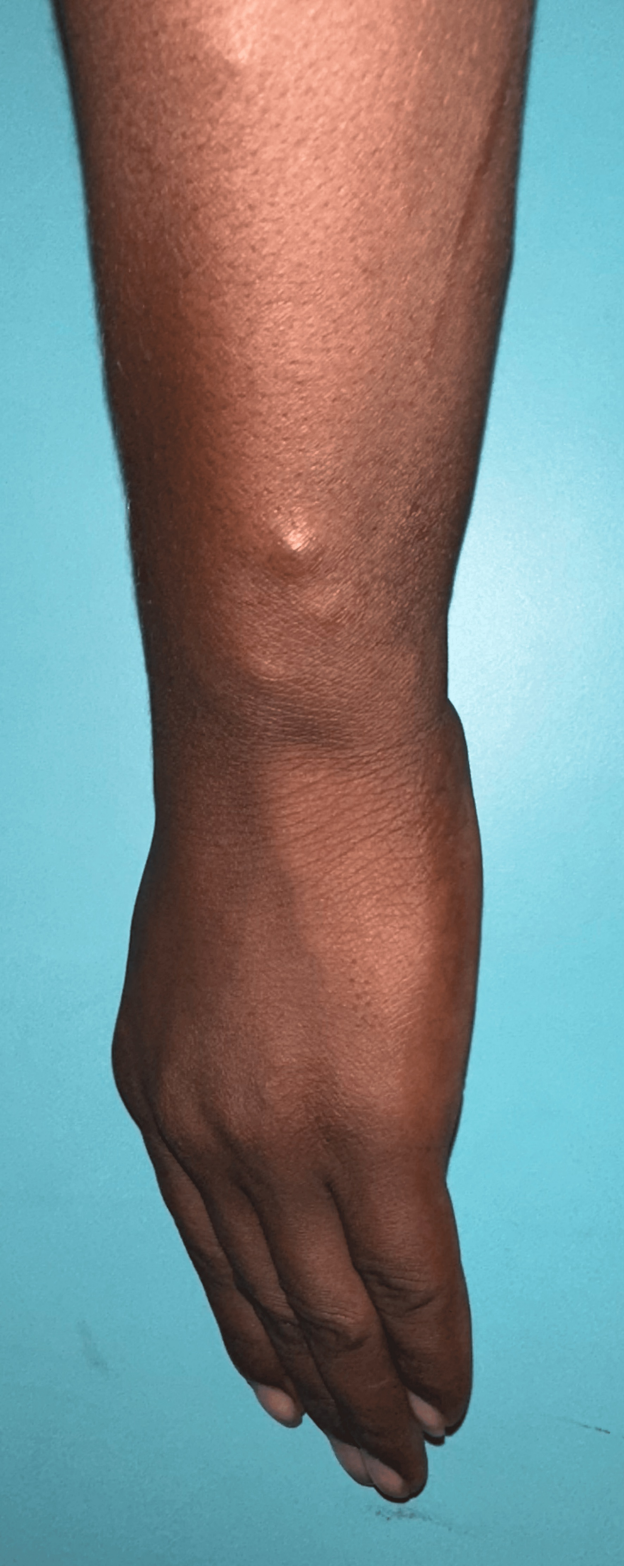
Skin-colored, non-tender subcutaneous nodules along the course of the lateral cutaneous nerves of the forearm

**Figure 2:**
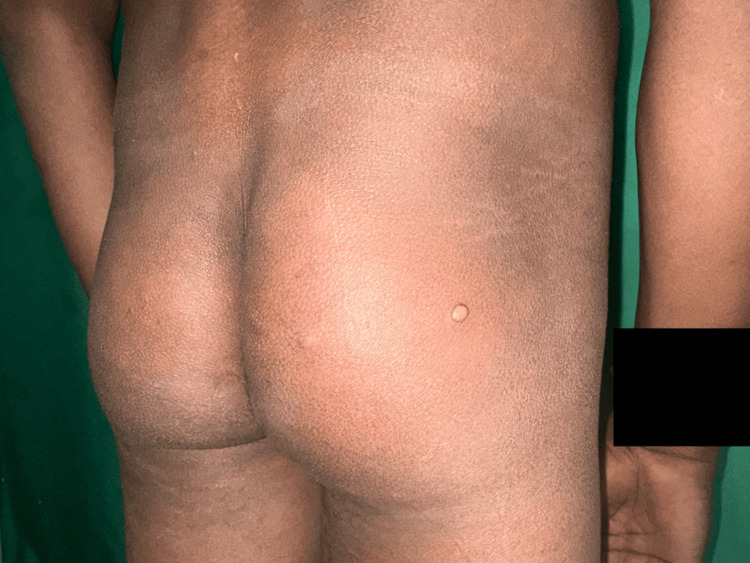
Pink-colored dermal nodule located on the buttock

**Figure 3:**
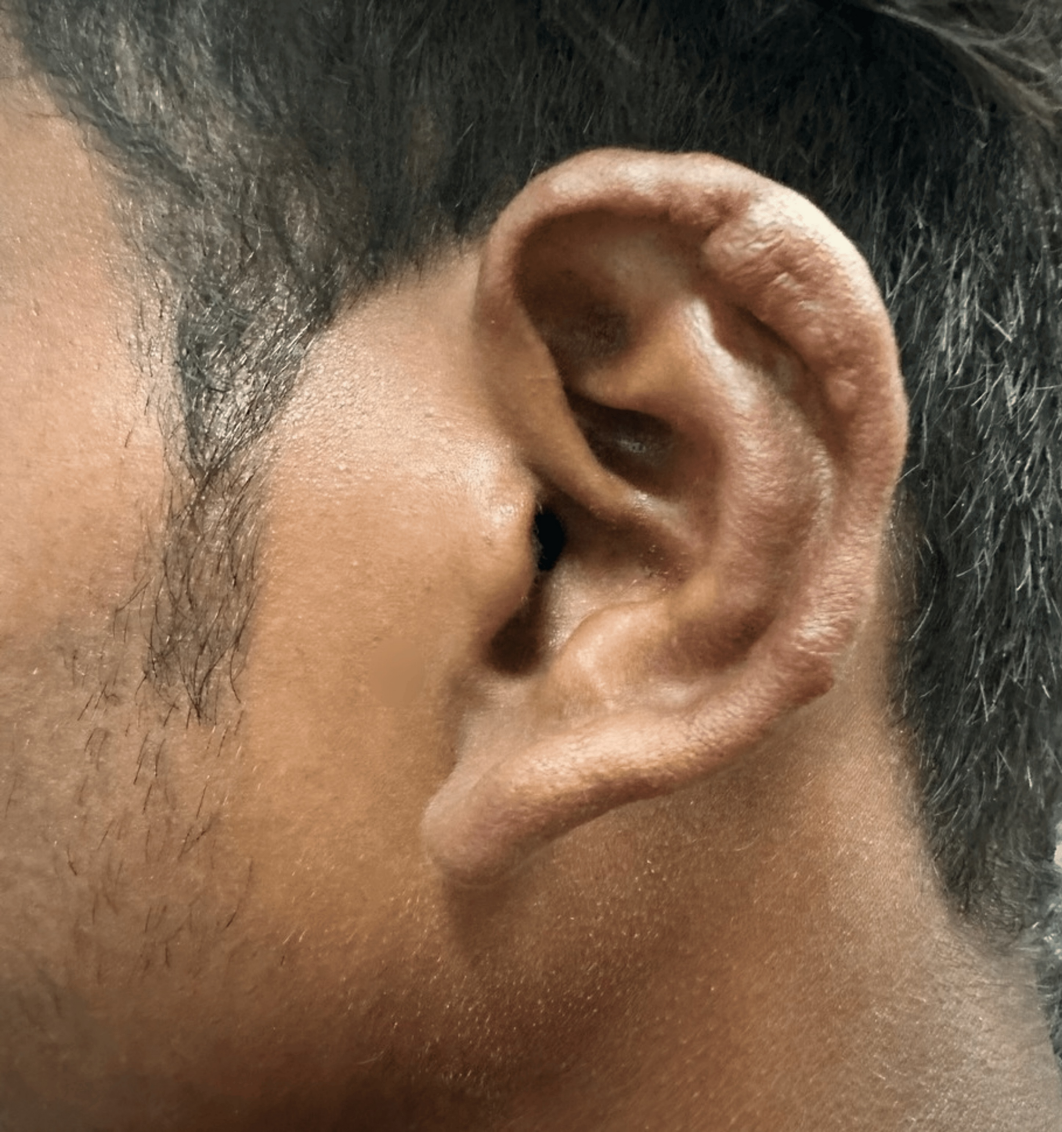
Nodular infiltration of the left ear

A 25-year-old male presented with a two-month history of painless nodules over the left forearm, right wrist, and right ankle joint. There was a mild yellowish tint to the nodules. On examination, there were five to six non-tender subcutaneous nodules attached to thickened superficial cutaneous nerves. The nerves associated were the lateral superficial cutaneous nerves of the left forearm, the right radial cutaneous nerve in the anatomical snuff box, and the right sural nerve near the Achilles tendon. The rest of the nerves examined were normal (Figure 1). Additionally, 10-15 firm, pink, shiny dermal nodules were scattered over the back, the buttocks, and the lower limbs (Figure 2). There were no associated sensory-motor deficits. He also had nodular thickening of the left earlobe (Figure 3), as well as multiple ill-defined hypopigmented macules with a slight coppery hue that were present symmetrically over the back. There was no associated sensory-motor loss. On further probing, the patient revealed that his mother had been a diagnosed and treated case of leprosy.

**Figure 4:**
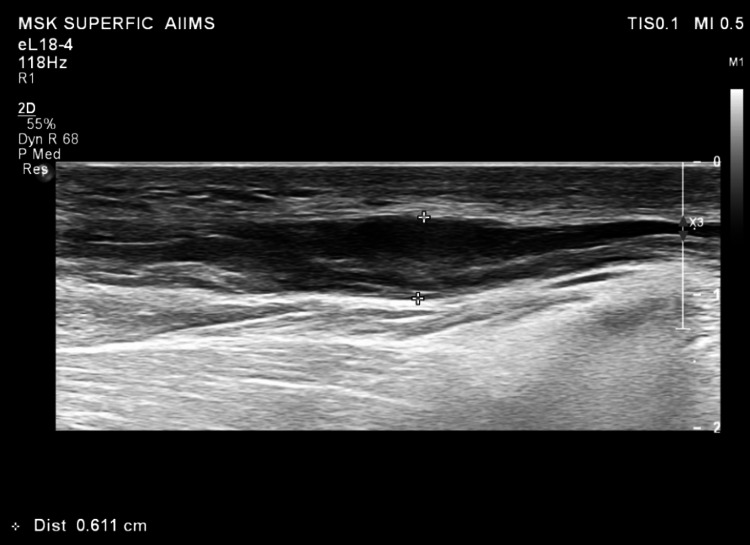
Longitudinal ultrasound view of swelling over the radial cutaneous nerve showing an avascular, heterogeneous hypoechoic lesion over a focally thickened nerve with loss of the normal fibrillar pattern

**Figure 5:**
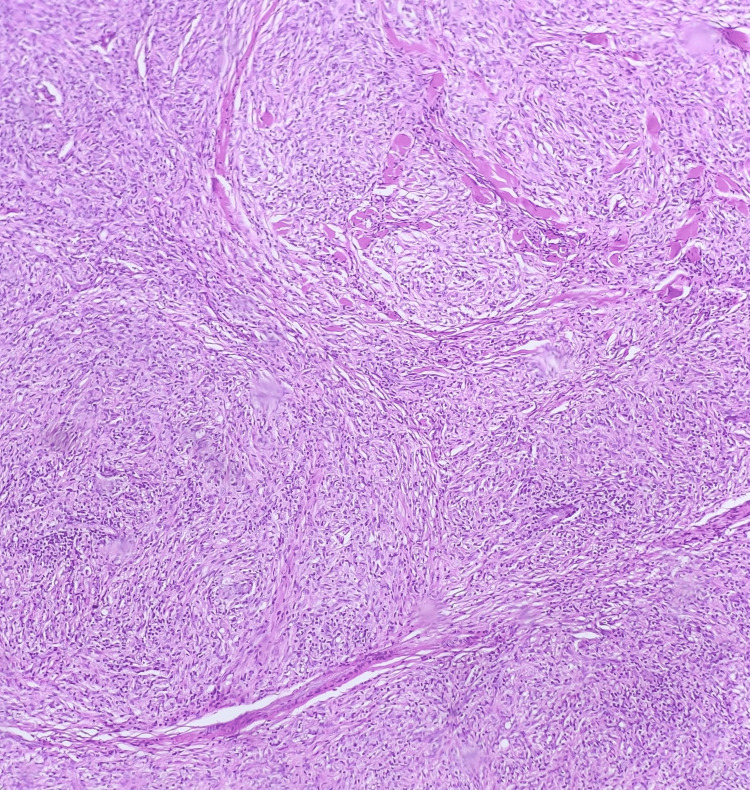
Well-circumscribed nodular and densely packed infiltration of spindle-shaped histiocytes arranged in a storiform to whorled pattern (H&E stain, 100× magnification).

**Figure 6:**
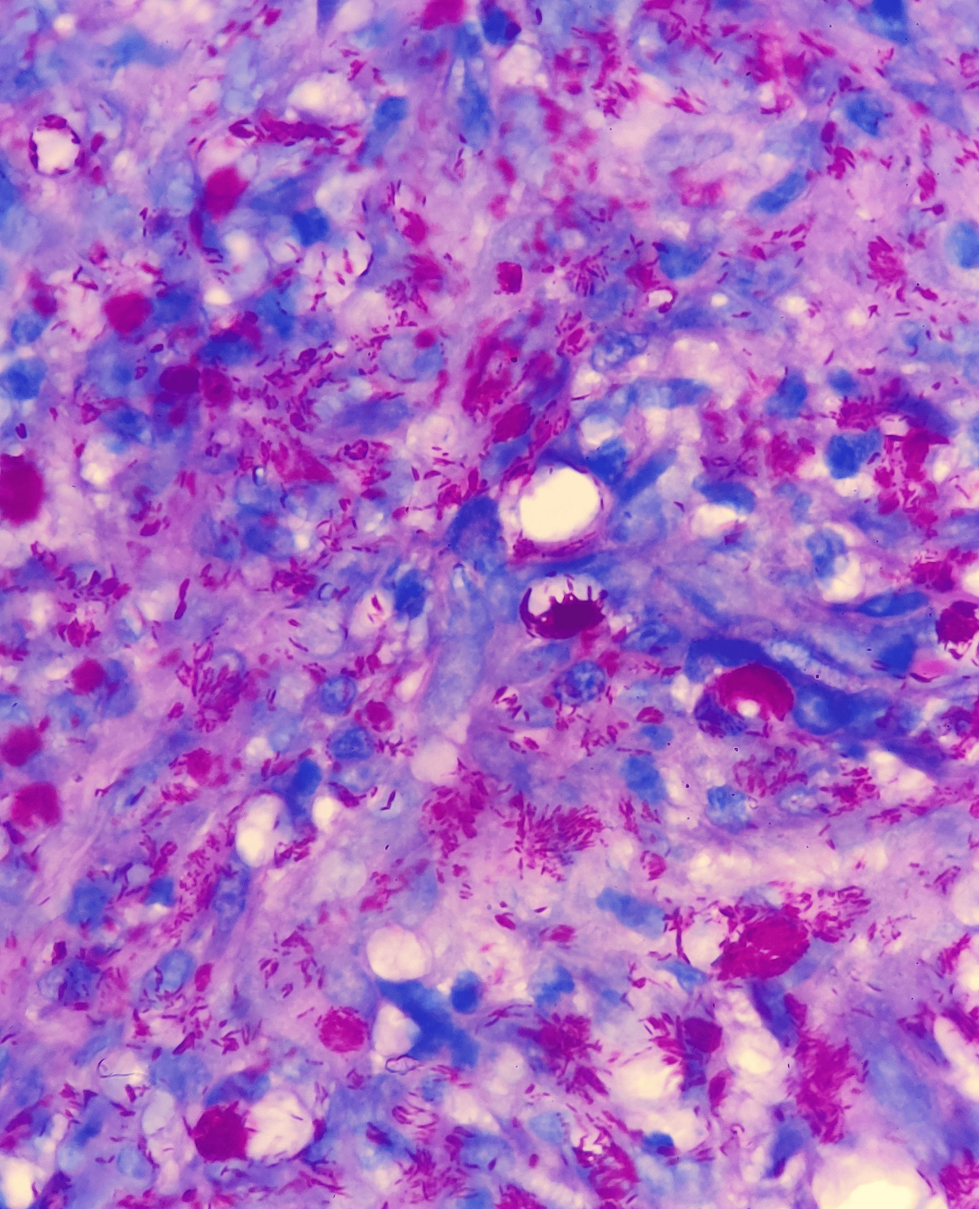
Numerous acid-fast bacilli within macrophages, some forming globi (Ziehl-Neelsen stain, 1000× magnification)

**Figure 7:**
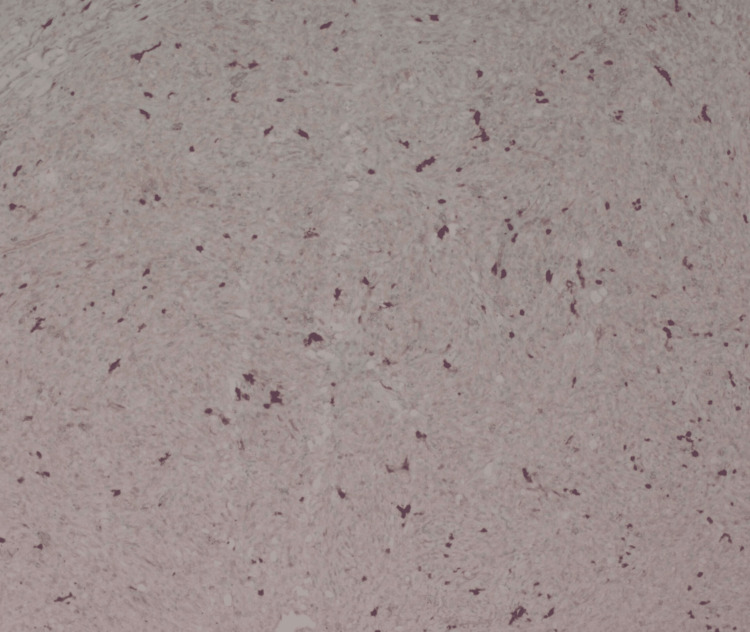
Immunohistochemistry showing faint positive staining for S100 protein (1000× magnification)

Ultrasonography of all of these subcutaneous nodules revealed well-defined, avascular, heterogenous, hypoechoic lesions over focally thickened nerves with loss of fibrillar pattern (Figure 4). The incisional biopsy of the lesion over the lateral aspect of the left forearm grossly revealed a rubbery, white, glistening nodule arising from a cutaneous nerve branch. It was carefully dissected out from the underlying nerve. Histopathology revealed well-circumscribed, nodular, and densely packed infiltration of spindle-shaped histiocytes with elongated nuclei and abundant eosinophilic cytoplasm. The histiocytes were arranged in a storiform to whorled pattern (Figure 5). Fite-Faraco stain was positive with numerous AFB within the histiocytes, some of which were forming globi. The bacteriological index was 6+ (Figure 6). S100 was faintly positive in the nodules (Figure 7). These findings were suggestive of histoid leprosy, which has a unique presentation as nodules arising from nerves.

Skin slit smears from normal-appearing skin over the forehead, buttock, and finger were 4+, 3+, and 4+, respectively, and 6+ from the subcutaneous nodule. The patient was started on WHO-recommended multibacillary multidrug therapy and showed clearing of lesions by the fourth month of follow-up. However, the patient was subsequently lost to follow-up.

## Discussion

Histoid leprosy is a rare and unusual presentation of leprosy with a high bacillary load. The original report described succulent, soft, expansile, pseudocapsulated, subcutaneous nodules. These nodules may migrate to the surface and fuse with the dermis to become elevated/protuberant [3]. They break down through a central softening process. Sometimes, molluscum-like papules and transepidermal material can happen at this stage. The lesions heal by distinctive scarring [4].

The histology is distinctive from ordinary leproma, featuring spindle-shaped histocytes with strands and whorls, which can be confused with spindle cell tumours. The expansile nature of histoid leprosy forms a pseudo-capsule, unlike the usual lepromas, which are infiltrative. Bacilli usually do not form globi and elongate in the direction of spindle cells. Some of the histoid lesions show tuberculoid foci with a lack of bacilli, which is termed tuberculoid contamination [3].

In this case, a storiform arrangement of histiocytes was observed, prompting consideration of differential diagnosis including schwannoma, dermal nerve sheath myxoma, soft tissue perineurioma, and other storiform or spindle cell tumours. However, none of these differentials have nodular thickening of the ear or copper hue macules. Considering this unique feature, along with the patient’s family history of leprosy, a Fite-Faraco stain was performed. This revealed numerous AFB within the spindle-shaped histiocytes, leading to the definitive diagnosis of neural histoid leprosy disease. Interestingly, in our case, some bacilli formed globi, which is uncommon in histoid leprosy.

Although previously believed to be confined to nodular lesions, newer evidence suggests that non-lesional skin in histoid leprosy can also harbor bacilli. In a series of six patients, non-lesional biopsies revealed foam cells, perineurovascular infiltrates, and bacillary presence without clear circumscription. This finding supports the hypothesis that histoid leprosy may represent a focalized manifestation due to some localized hyperimmune response in cases with underlying lepromatous disease [5]. In our case, there was clear evidence of lepromatous disease with the presence of coppery-hued macules and positive skin slit smears from clinically uninvolved skin.

Several unusual varieties of histoid leprosy are also reported, including mucosal histoid [6], keloidal [7], large tumor-like lesions [8], figurate erythema-like [9], and xanthogranuloma-like [10]. Few cases of histoid leprosy arising from nerve are reported in the literature, either as a case of relapse [11] or as de-novo [12]. To our knowledge, no new case has been reported for three decades. Our case adds to the growing body of literature on atypical histoid leprosy.

The etiology of histoid transformation remains incompletely understood. It has been postulated to result from focal immune suppression or the clonal selection of drug-resistant bacilli in localized skin regions [11]. The cell-mediated immunity is found to be relatively high in patients with histoid leprosy compared to patients with lepromatous leprosy, albeit less than that in normal patients [13]. An alternative hypothesis proposes a hyperimmune response in an effort to restrict/localize or focalize the disease [14].

Our case introduces a rare and biologically unique feature: histoid nodules arising directly from peripheral nerves. *Mycobacterium leprae *specifically targets Schwann cells, causing epigenetic and transcriptional changes, leading to their reversion into a progenitor-like dedifferentiated state, which can migrate, allowing bacterial dissemination to various tissues, and then redifferentiate into the mesenchymal lineage. It is possible that due to still unknown factors, these Schwann cells show localized hyperproliferation without migration, appearing as spindle cells with mesenchymal differentiation [15]. It is plausible that in our case, histoid cells were derived from the Schwann cells, as demonstrated by low positive S100 staining in our case.

## Conclusions

Histoid leprosy can present with various atypical and difficult-to-diagnose forms. Recognizing myriad presentations of histoid leprosy might help in early diagnosis and management. The neural origin of these nodules also supports the theory that Schwann cells re-differentiate into mesenchymal cells in histoid leprosy.
